# Rhizosphere microbial diversity analysis reveals enrichment of biocontrol taxa in healthy tobacco plants and yields novel antagonists against *Phytophthora nicotianae*

**DOI:** 10.3389/fpls.2026.1889242

**Published:** 2026-09-07

**Authors:** Juan Xu, Wenqiang Mei, Junli Shi, Juan Li, Ming Liu, Zhijuan Yang, Nengfei Tian, Yanxia Hu

**Affiliations:** 1 Dali Prefecture Branch of Yunnan Tobacco Company, Dali, China; 2 College of Pharmaceutical Science, Dali University, Dali, China; 3 Yunnan Academy of Tobacco Agricultural Sciences, Kunming, China; 4 Dali Branch of Dali State Tobacco Company, Dali, China; 5 College of Plant Protection, Yunnan Agricultural University, Kunming, China

**Keywords:** antagonistic bacteria, biological control, phytophthora nicotianae, rhizosphere microbiota, tobacco black shank

## Abstract

**Introduction:**

Tobacco black shank (TBS), caused by *Phytophthora nicotianae*, is a devastating disease. Current control relies heavily on chemical pesticides, which pose risks of resistance and environmental pollution. Biological control using rhizosphere microorganisms offers a sustainable alternative. However, the relationship between the composition of the rhizosphere microbiome and its suppressive activity against *P. nicotianae* has not yet been systematically elucidated.

**Methods:**

High-throughput sequencing was used to compare the differences in rhizosphere microbiota of healthy and diseased plants from multiple field sites in Yunnan, China, combined with culture-dependent isolation and screening of antagonistic microorganisms against *P. nicotianae*.

**Results:**

No significant differences in bacterial or fungal diversity were found between healthy and diseased rhizosphere soils. Despite this overall similarity in diversity, healthy rhizosphere soil was significantly enriched in biocontrol genera, including *Pseudomonas*, *Paenibacillus*, and *Massilia*. A total of 204 bacterial and 95 fungal strains were isolated, among which 64 bacteria and 41 fungi exhibited antagonistic activity against *P. nicotianae*. Four strains (710, 733, 1088, and 1145) showed the strongest inhibitory effects and were identified as *Bacillus amyloliquefaciens*, *Yokenella* sp., *Metapseudomonas resinovorans*, and *Bacillus subtilis*, respectively. Moreover, the soluble metabolites and volatile metabolites produced by these strains also inhibited *P. nicotianae*. Notably, this study provides the first evidence that *Y. regensburgei* and *M. resinovorans* function as biocontrol agents against *P. nicotianae*, with mycelial inhibition rates reaching 72.29% and 38.96%, respectively.

**Discussion:**

These results reveal the enrichment of specific biocontrol taxa in healthy tobacco rhizosphere and identify *Y. regensburgei* and *M. resinovorans* as novel antagonists against *P. nicotianae*. This study expands the strain library for TBS biological control and provides promising candidates for developing green management strategies.

## 1 Introduction

Tobacco (*Nicotiana tabacum* L.) is a major economic crop worldwide, serving as a significant source of agricultural income in many countries (Wang et al., 2022). However, its production is severely restricted by various diseases, among which tobacco black shank (TBS) caused by *Phytophthora nicotianae* is one of the most destructive. This disease leads to substantial yield losses and quality reduction, imposing heavy economic burdens on tobacco farmers and related industries (Yu et al., 2024; Zhu et al., 2024). Current management strategies for TBS mainly rely on chemical pesticides, crop rotation, and the selection of disease-resistant varieties. However, these approaches suffer from inconsistent efficacy, environmental pollution, and pesticide residues in tobacco leaves (Guo et al., 2020; Li J, et al., 2023). Thus, there is an urgent need to develop sustainable and environmentally friendly strategies for TBS control.

Rhizosphere microorganisms serve as the first line of defense for plants, with their community composition, diversity, and interaction network closely linked to plant disease resistance (Berendsen et al., 2012; Mendes et al., 2013). Numerous studies have demonstrated that beneficial rhizosphere microorganisms can effectively inhibit various soil-borne diseases. For example, *Flavobacterium* TRM1 isolated from tomato rhizosphere suppresses *Ralstonia solanacearum* (Kwak et al., 2018), and *Bacillus velezensis* K0T24 achieves 55.17% control efficacy against mulberry bacterial wilt (Jiao et al., 2024). These findings highlight the potential of rhizosphere-derived biocontrol agents as a promising and sustainable alternative for mitigating soil-borne diseases.

The mechanisms by which beneficial microorganisms suppress soil-borne diseases primarily include direct antagonism and indirect induction. Direct antagonism involves the production of antimicrobial compounds, such as lipopeptide antibiotics and volatile organic compounds, competition for ecological niches and nutrients, and degradation of pathogen cell walls via secreted extracellular enzymes (Gómez Expósito et al., 2017; Niu et al., 2020). Indirect mechanisms mainly operate through the induction of plant systemic resistance, activating defense-related enzymes and defense-related gene expression, thereby enhancing plant resistance against pathogen infection (Pieterse et al., 2014; Wu et al., 2023). Several antagonistic mechanisms have been reported specifically for *P. nicotianae*. For instance, *Bacillus velezensis* EM-1 effectively inhibits *P. nicotianae* growth by secreting volatile compounds, lipopeptides, and polyketide metabolites (Sui et al., 2022). *B. subtilis* GXHZ16 restricts *P. nicotianae* by upregulating genes associated with flagellar assembly, chemotaxis, and the synthesis of surfactin and fengycin (Hu et al., 2024). *B. velezensis* SDTB022 enhances tobacco resistance against TBS by stimulating the expression of defense-related genes (e.g., *PR1a*, *PR3*, and *PR5*) and boosting the activities of key defense enzymes such as polyphenol oxidase, peroxidase, and phenylalanine ammonia-lyase (Qiu et al., 2022). Collectively, these findings demonstrate that rhizospheric beneficial microbes inhibit *P. nicotiana*e through multiple synergistic mechanisms, providing a robust theoretical basis for the development of stable biocontrol agents.

Notably, TBS is also a typical soil-borne disease, and its pathogen *P. nicotianae*, can survive in the soil for extended periods. Previous studies have reported that TBS infection disrupts the homeostasis of the tobacco rhizosphere microbial community, and under disease stress, tobacco roots recruit beneficial microorganisms to resist pathogen invasion (Ma et al., 2025). Therefore, isolating and screening antagonistic strains with high inhibitory activity against *P. nicotianae* from tobacco rhizosphere soil is a feasible and effective approach for TBS biological control. Currently, antagonistic microorganisms effective against TBS are mainly from common genera, such as *Bacillus* sp (Ding et al., 2021; Tao et al., 2025), *Pseudomonas* sp (Ma et al., 2024; Tao et al., 2025), *Talaromyces* sp (Tian et al., 2020; Tao et al., 2025), *Chaetomium* sp (Wang et al., 2026), *Trichoderma* sp (Liu et al., 2022; Yin et al., 2025), and *Streptomyces* sp (Huang et al., 2015; Zhu et al., 2026). However, the exploration and utilization of novel and efficient antagonistic microbial resources in the tobacco rhizosphere remain insufficient, which seriously hinders the further development and practical application of sustainable biological control strategies against TBS. Thus, isolating and identifying diverse anti-TBS bacteria and fungi with strong disease-suppressive effects is crucial for expanding the biocontrol strain library and developing stable, effective composite microbial agents.

To address these knowledge gaps, this study aimed to (i) characterize the diversity, composition, and functional profiles of rhizosphere microbial communities associated with healthy and TBS-infected tobacco plants, thereby clarifying how rhizosphere microbial characteristics influence TBS development; and (ii) isolate culturable strains with strong antagonistic activity against *Phytophthora nicotianae* and evaluate the inhibitory effects of their soluble and volatile metabolites. This study provides valuable microbial resources and a scientific basis for the sustainable biocontrol of TBS.

## 2 Materials and methods

### 2.1 Soil information and sample collection

A total of 252 rhizosphere soil samples were collected from 42 fields across 12 counties in Dali Prefecture, Yunnan Province. Using a paired sampling design, three healthy and three diseased samples were collected from each field (**Supplementary Table 1**). Each sample was independently processed as a biological replicate. Disease severity was assessed according to previously reported standards (Gao et al., 2015): healthy plants (grade 0) showed no disease symptoms, whereas diseased plants (grade 7) exhibited stem lesions encircling the entire stem or wilting of more than two-thirds of the leaves. For rhizosphere soil collection, the upper 10-15 cm of topsoil was first removed, after which roots at a depth of 20 cm were carefully excavated using a sterile shovel. After removing large soil aggregates, soil tightly adhering to fine roots (1-2 mm in diameter) was gently brushed off and collected as rhizosphere soil (Yang et al., 2023).

### 2.2 Analysis of microbial diversity in tobacco rhizosphere soil

#### 2.2.1 DNA extraction and Illumina sequencing

DNA was extracted from 0.5 g of soil (wet weight) using the E.Z.N.A.^®^ soil DNA kit (Omega Bio-Tek, Norcross, GA, USA) following the manufacturer’s protocol. The DNA concentration was measured with a Nanodrop 2000 (Thermo Scientific, Waltham, MA, U.S.), and its quality was assessed by 1.0% agarose gel electrophoresis. The OD_260/280_ ratio of the extracts ranged from 1.8 to 2.0. Fungal and bacterial DNA were amplified separately. For fungi, the ITS1 (5’-TCCGTAGGTGAACCTGCGG-3’) and ITS2 (5’-GCTGCGTTCTTCATCGATGC-3’) primers were used. For bacteria, the V4-V5 region was amplified with primers 515F (5’-barcode-GTGCCAGCGCCGG-3’) and 907R (5’-CCGTCAATTCMTRATTTT-3’). Each 20 µL PCR reaction contained 10 ng DNA template, 4 µL 5× FastPfu buffer, 2.5 µL 2.5 mM dNTPs, 0.8 µL of each 5 μM, 0.4 µL FastPfu Polymerase, and ddH_2_O. The amplification program was: 95°C for 5 min, followed by 30 cycles (denaturation at 95°C for 30 s, annealing at 58°C for 30 s, extension at 72°C for 45 s), and finally extension at 72°C for 10 min. PCR products were separated on 2% agarose gels, purified using the AxyPrep DNA Gel Extraction Kit (Axygen Biosciences, Union City, CA, U.S.), and quantified with Qubit^®^3.0 (Life Invitrogen). High-throughput sequencing was performed on the Illumina HiSeq 2500 PE250 platform.

#### 2.2.2 Processing of sequencing data

Low-quality sequences were removed using QIIME, and chimeric sequences were filtered out with Usearch to obtain high-quality reads. Operational taxonomic units (OTUs) were clustered at 97% similarity using UPARSE. Taxonomic classification of representative OTU sequences was performed with the RDP Classifier (version 2.2) against the Silva database (16S rRNA) and the UNITE database (ITS), respectively.

#### 2.2.3 Bioinformatics and data analysis

Rarefaction analysis was conducted using Mothur v.1.21.1 (Schloss et al., 2009) to calculate α-diversity indices (Chao1, ACE, Simpson, and Shannon). Differences in α-diversity were assessed using the Kruskal-Wallis or Mann-Whitney U test. For β diversity, principal component analysis (PCA) was performed based on Euclidean distance, while principal co-ordinates analysis (PCoA) and Non-metric Multidimensional Scaling (NMDS) were based on Bray-Curtis (Xiong et al., 2020; Luo et al., 2021). Biomarkers were identified using linear discriminant analysis (LDA) effect size (LEfSe) with an LDA score threshold of 2. Clustering heatmaps were generated using R (v3.6.0). Co-occurrence networks were constructed using the WGCNA package based on Spearman’s correlation matrices (Spearman’s *r* > 0.8, p-value < 0.05) from the 16S rRNA sequencing data (Chen et al., 2022). Functional predictions of microbial communities were performed using Phylogenetic Investigation of Communities by Reconstruction of Unobserved States (PICRUSt2) (Chen et al., 2022) based on the KEGG database.

### 2.3 Determination of soil physicochemical properties

Soil physicochemical properties were determined as follows: pH was measured in a 1:2.5 soil-to-water suspension; organic matter (OM) by potassium dichromate oxidation; hydrolyzable nitrogen (HN) by alkali hydrolysis diffusion; available phosphorus by molybdenum–antimony colorimetry; available potassium by ammonium acetate extraction–flame photometry; total nitrogen (TN) by the Kjeldahl method; total phosphorus (TP) by alkali fusion–molybdenum–antimony colorimetry; total potassium (TK) by sodium hydroxide fusion–flame photometry; and available boron (AB) by azomethine-H spectrophotometry.

### 2.4 Isolation and identification of cultivable microorganisms in the tobacco rhizosphere soil

#### 2.4.1 Isolation of cultivable microorganisms

The cultivable rhizosphere bacteria and fungi were isolated as described previously with minor modifications (Yi et al., 2022; Krstić Tomić et al., 2023). A 5 g soil sample from each region was weighed and mixed with 45 mL of sterile water. The mixture was incubated with shaking at 28°C and 180 r/min for 24 h to prepare the microbial suspension. Then, 1 mL of the suspension was diluted tenfold with sterile water to a dilution of 10^-5^ or 10^-6^. For bacteria, 200 μL aliquots from each dilution were plated onto LB agar plates. For fungi, 200 μL of each dilution was spread onto PDA plates. The plates were incubated at 28°C for 2–4 days. The colonies with different morphologies were selected and purified. Each microbial species was preserved in 30% glycerol (v/v) at −80°C.

#### 2.4.2 Molecular biological identification of strains

Total DNA of pure cultured bacteria and fungi was extracted using the Bacterial genomic DNA extraction kit (Tiangen Biochemical Technology Co., Ltd., Beijing, China) and the plant genomic DNA extraction kit (Tiangen Biochemical Technology Co., Ltd., Beijing, China), respectively. For bacteria, primers 27F (5′-AGAGTTTGATCATGGCTCAG-3′) and 1492R (5′-TACGGCTACCTTGTTACGACTT-3′) were used; for fungi, primers ITS1F (5′-CTTGGTCATTTAGAGGAAGTAA-3′) and ITS4R (5′-TCCTCCGCTTATTGATATGC-3′) were employed. The PCR products were purified and sequenced at the Chinese Academy of Agricultural Sciences. The obtained sequences were subjected to BLAST alignment in the NCBI database (https://www.ncbi.nlm.nih.gov/) to enable the preliminary classification and identification of the strains.

#### 2.4.3 Phylogenetic analysis

Based on the BLAST results from section 2.3.2, reference sequences of type strains or well−characterized species with high similarity to the query sequences were retrieved from the NCBI GenBank database. Both the query sequences and the reference sequences were trimmed using MEGA6 (Tamura et al., 2013) to remove low-quality ends. Multiple sequence alignments were conducted using the MAFFT (Katoh and Standley, 2013) online server (https://mafft.cbrc.jp/alignment/server/index.html, accessed on 31 March 2026). A phylogenetic tree was constructed using the maximum−likelihood (ML) method implemented in MEGA6. Bootstrap analysis with 1000 replicates was performed to assess the robustness of tree topologies. The resulting tree was visualized and annotated using MEGA6.06 or Chiplot (Xie et al., 2023) (https://www.chiplot.online/).

### 2.5 Screening of antagonistic microorganisms against *P. nicotianae*

According to previous methods (BiBi et al., 2023), the plate confrontation method was used to screen for antagonistic bacteria and fungi against *P. nicotianae*. For bacteria assay, first, each test strain was inoculated into LB liquid medium and cultured at 28 °C with shaking at 180 r/min for 24 h. Subsequently, *P. nicotianae* plug (5 mm in diameter) was placed at the center of a PDA plate. Then, 50 μL of the test bacterial suspension (OD_600_ = 0.8) was streaked on both sides of the plug, maintaining a 30 mm distance. For the fungus assay, test fungal plugs of the same size as the *P. nicotianae* plug were inoculated on both sides of the PDA plate, maintaining a 30 mm distance. Sterile water was used as a negative control. The inhibition zone was observed and recorded after incubating the plates at 28°C in the dark for 3–5 days. All treatments were performed in triplicate. Four strains exhibiting strong antagonistic effects were identified in the initial screening. Their suspension concentrations were adjusted to the same OD_600_ value, and the antagonistic assay was repeated following the aforementioned procedure. On day 7, the vertical and horizontal diameters of the pathogen colony were measured, and the inhibition rate was calculated.

### 2.6 Inhibitory effects of bacterial soluble metabolites on *P. nicotianae*

The sandwich agar plate method (Zhang J et al., 2024) was used to evaluate the inhibitory effect of the soluble metabolites of the strain on *P. nicotianae*. Briefly, the bacterial strains were first cultured in LB liquid medium at 37°C and 180 r/min for 24 h. Then, 100 μL of the same standardized bacterial suspension (OD_600_ = 0.8) was inoculated onto LB solid medium, which was then covered with PDA solid medium for inoculating *P. nicotianae*. The plates were subsequently incubated at 28°C in a constant-temperature incubator. After 10 days of incubation, the diameter of the fungal colony was measured, and the inhibition rate was calculated using the following formula: Inhibition rate (%) = (Control colony diameter-Treated colony diameter)/Control colony diameter × 100.

### 2.7 Inhibitory effects of bacterial volatile metabolites on *P. nicotianae*

The donut plate method (Xu et al., 2022) was employed to examine the effects of bacterial volatiles on the fungus *P. nicotianae*. Specifically, 5-mm-diameter mycelial plugs were placed at the center of a 60-mm Petri dish containing PDA medium. This inner dish was then placed within a larger 90-mm Petri dish to create a separated co-culture system. In the outer compartment, LB medium was added, and subsequently inoculated with 20 μL of the same standardized bacterial suspension (OD_600_ = 0.8) after a 24-hour pre-cultivation period (at 37°C, 180 rpm). The co-culture was incubated at 28°C, and after six days, the diameter of the fungal colony was measured to assess the influence of bacterial volatiles on fungal growth.

### 2.8 Statistical analysis

All experimental data were replicated three times. Statistical analyses were conducted using a t-test in IBM SPSS Statistics 26.0 (Chicago, IL, USA), and graphical representations were generated with GraphPad Prism 8 software (San Diego, CA, USA). A p-value < 0.05 was considered statistically significant.

## 3 Results

### 3.1 Sequencing data and OTU clustering

**Figure 1:**
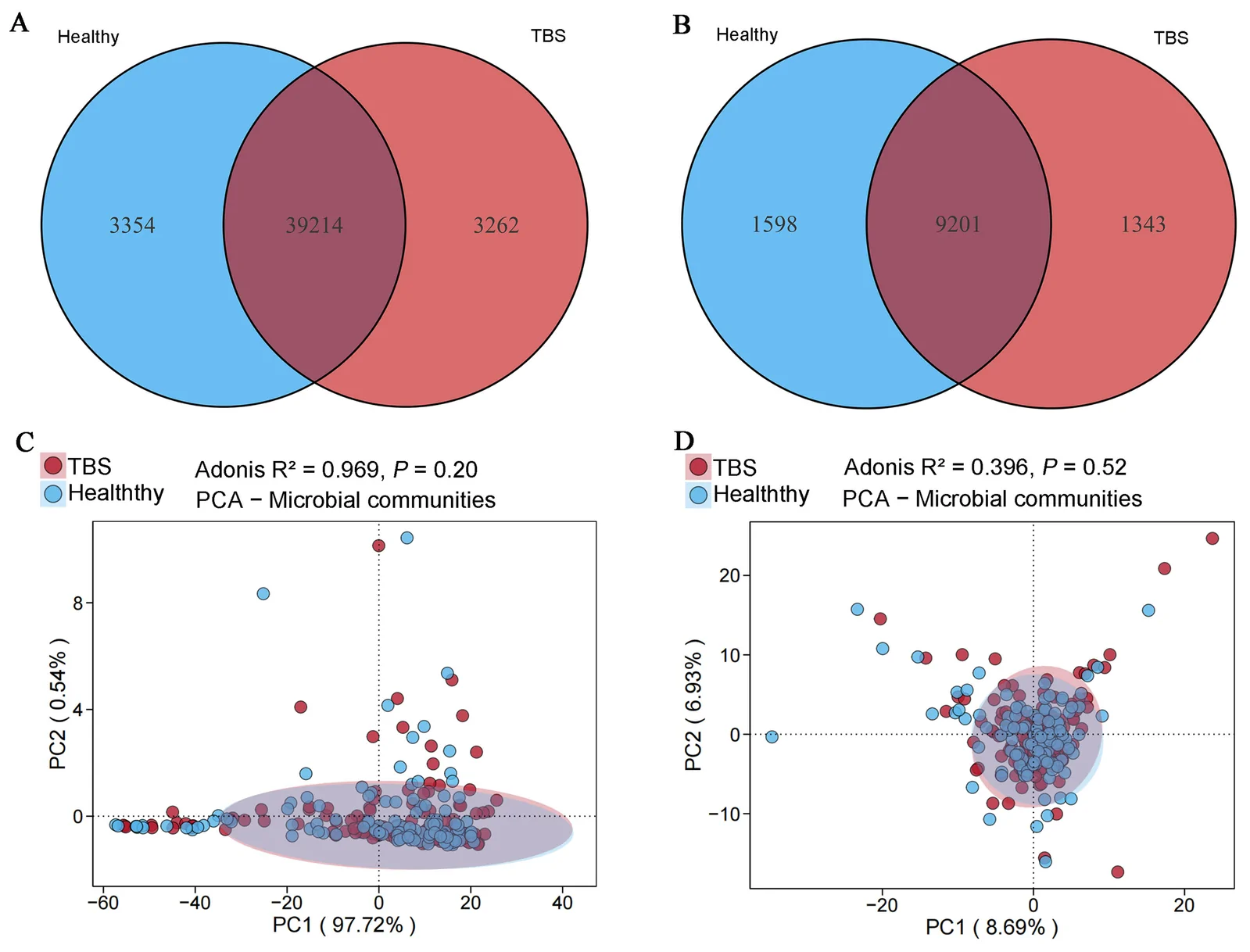
OTU variance analysis of microbes in the rhizosphere soil of healthy and TBS-infected tobacco. Venn diagram of bacteria **(A)** and fungi **(B)**. PCA analysis of the bacterial **(C)** and fungal **(D)** community.

After using high-throughput sequencing technology to filter low-quality reads, barcodes, and primer sequences, a total of 15,660,528 high-quality 16S sequences (for bacteria) and 15,057,688 high-quality ITS sequences (for fungi) were obtained from 252 samples for subsequent analysis. To facilitate community characterization, non-redundant sequences (excluding singletons) from each dataset were clustered into OTUs at a 97% similarity threshold. For the bacterial community, the healthy and TBS groups yielded a total of 45,830 OTUs, with the healthy group and TBS group containing 42,658 and 42,476 OTUs, respectively. Among these, 3,354 OTUs were unique to the healthy group, while 3,262 OTUs were unique to the TBS group (**Figure 1A**). PCA revealed that the two groups clustered closely together (**Figure 1C**), indicating that the rhizosphere bacterial composition of diseased tobacco maintained a high degree of similarity to that of healthy plants. For the fungal community, a total of 12,142 OTUs were obtained, with the healthy and TBS groups containing 10,799 and 10,544 OTUs, respectively. The number of OTUs unique to the healthy and TBS groups was 1,598 and 1,343, respectively (**Figure 1B**). PCA showed no distinct separation between the healthy and TBS groups (**Figure 1D**), suggesting that the rhizosphere fungal communities of both diseased and healthy plants shared a similar composition.

### 3.2 Diversity analyses of the healthy and TBS-infected tobacco rhizosphere microbial community

**Figure 2:**
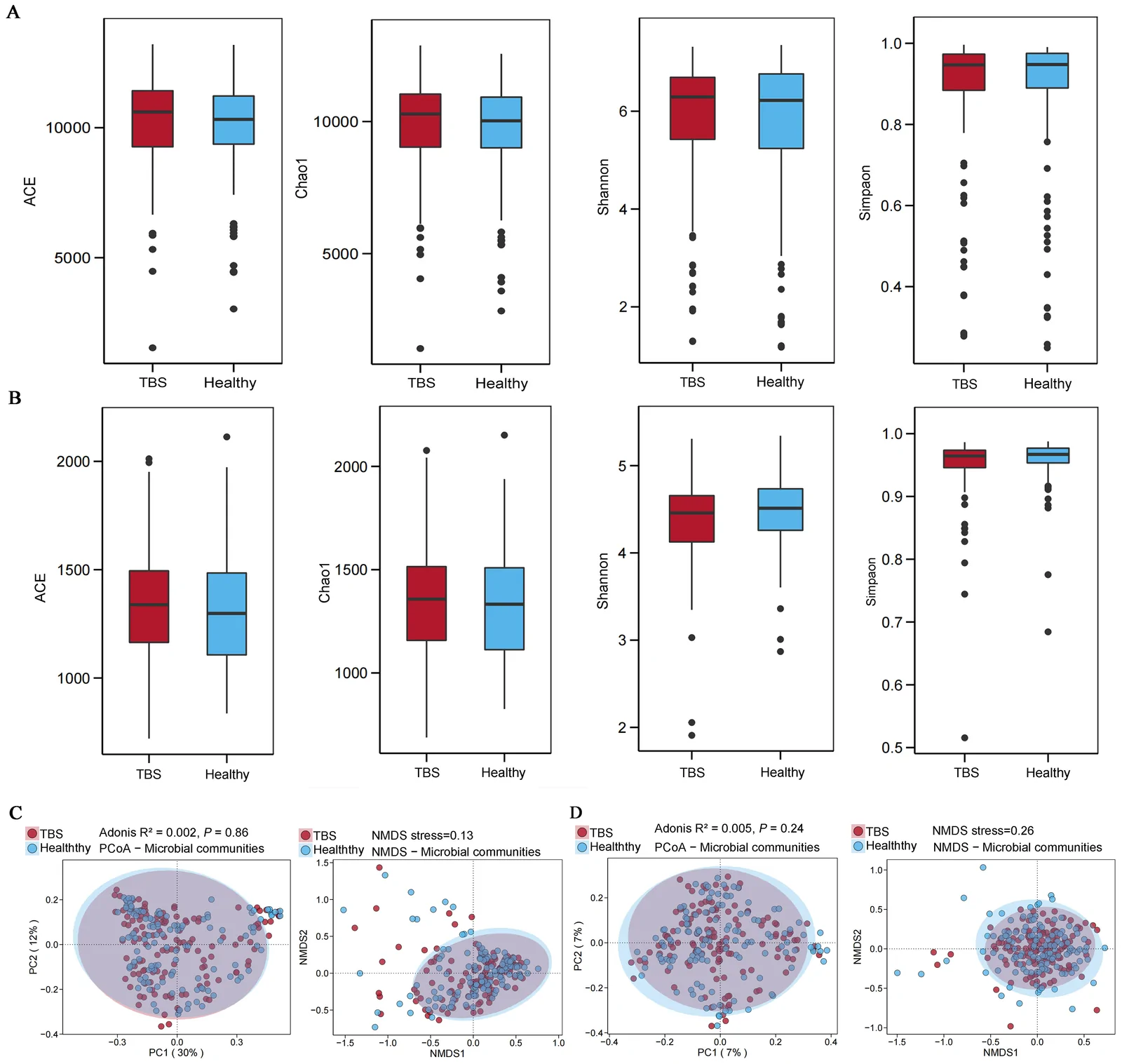
Diversity analyses of the healthy and TBS-infected tobacco rhizosphere microbial community. Four alpha diversity (ACE, Chao1, Shannon, and Simpson) indices of rhizosphere bacteria **(A)** and fungi **(B)**. PCoA and NMDS analysis of rhizosphere bacteria **(C)** and fungal **(D)** community.

To evaluate microbial diversity within and between groups, alpha diversity indices (ACE, Chao1, Shannon, and Simpson) were calculated. Results indicated that there were no significant differences between the Healthy and TBS groups for either bacteria or fungi (*P* > 0.05, **Figures 2A, B**). In addition, PCoA and NMDS based on the Bray-Curtis distance showed that the bacterial and fungal communities of the two groups clustered tightly together (**Figures 2C, D**). These findings further support the observation of a similar microbial composition between the healthy and TBS groups.

### 3.3 Composition of the healthy and TBS-infected tobacco rhizosphere microbial community

**Figure 3:**
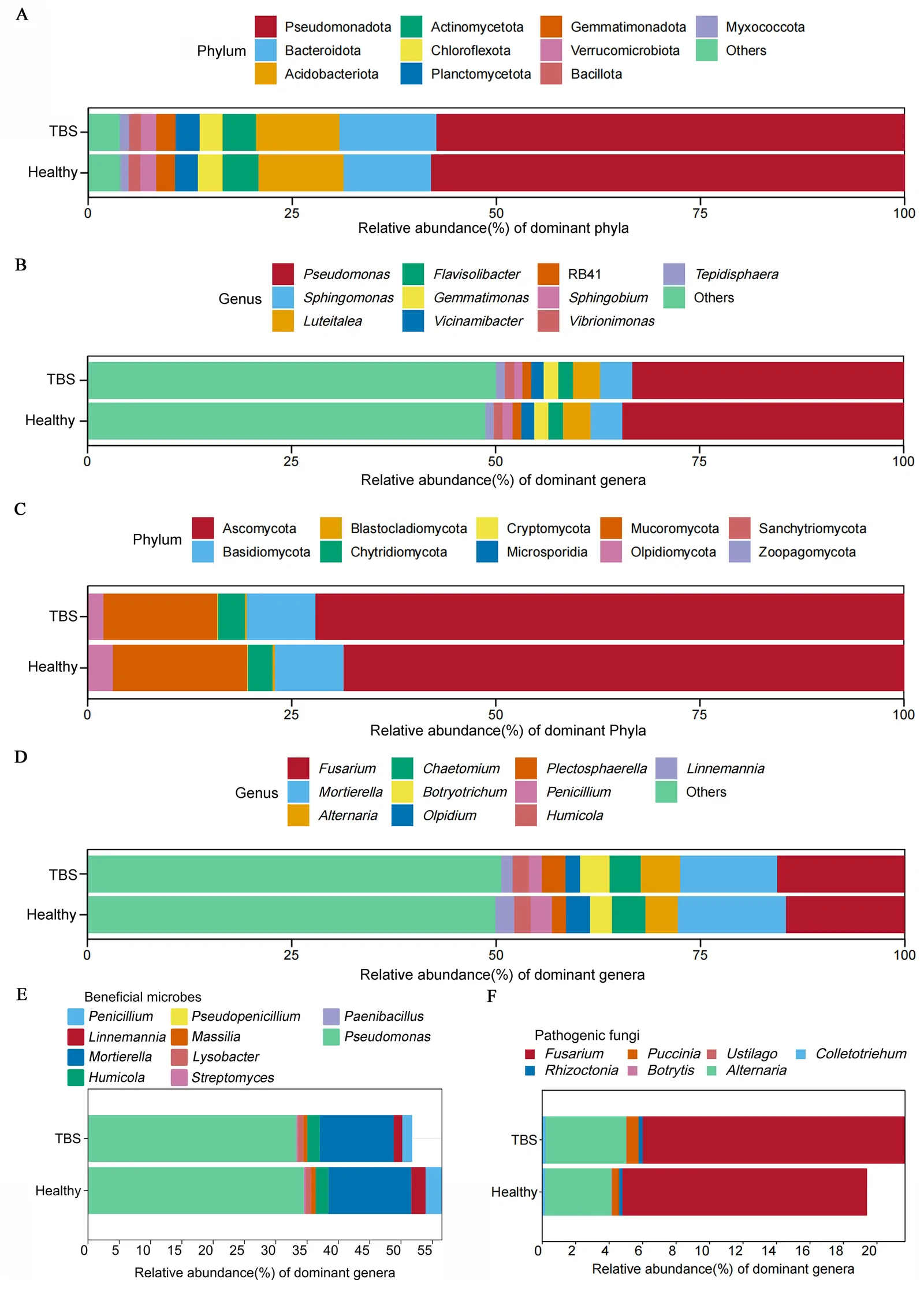
Characteristics of bacterial communities compositions at the phylum **(A)** and genus **(B)** level in healthy and TBS groups. Characteristics of fungal communities compositions at the phylum **(C)** and genus **(D)** level in healthy and TBS groups. Comparative analysis of rhizosphere beneficial microbes **(E)** and plant pathogenic fungi **(F)** in healthy and TBS groups.

To further investigate the changes in rhizosphere microbial community composition associated with TBS, this study compared the relative abundance of bacterial and fungal taxa at the phylum and genus levels between healthy and TBS-infected samples. At the bacterial phylum level, the top 10 bacterial taxa in both groups were Pseudomonadota, Bacteroidota, Acidobacteriota, Actinomycota, Chloroflexota, Planctomycota, Gemmatimonadota, Verrucomicrobiota, Bacillota, and Myxococcota. In the TBS group, the relative abundances of Pseudomonas, Acidobacteriota, Actinomycota, Chloroflexota, Verrucomicrobiota, and Bacillota decreased by 1.16%, 2.38%, 6.29%, 4.76%, 2.83%, and 0.92%, respectively. In contrast, Bacteroidota, Planctomycota, Gemmatimonadota, and Myxococcota increased by 11.03%, 2.48%, 4.45%, and 7.36%, respectively, compared with the healthy group (**Figure 3A**). At the bacterial genus level, the top 10 taxa included *Pseudomonas*, *Sphingomonas*, *Luteitalea*, *Flavisolibacter*, *Gemmatimonas*, *Vicinamibacter*, *Vibrionimonas*, RB41, *Tepidisphaera*, and *Sphingobium*. In the TBS group, the relative abundances of *Sphingomonas*, *Flavisolibacter*, *Gemmatimonas*, *Vibrionimonas*, and *Tepidisphaera* increased by 0.79%, 5.96%, 4.36%, 2.07%, and 3.77%, respectively, whereas *Pseudomonas*, *Luteitalea*, *Vicinamibacter*, RB41, and *Sphingobium* decreased by 3.51%, 3.39%, 4.47%, 4.65%, and 11.83%, respectively, compared with the healthy group (**Figure 3B**).

At the fungal phylum level, the top 10 taxa in both groups were Ascomycota, Basidiomycota, Blastocladiomycota, Chytridiomycota, Cryptomycota, Microsporidia, Mucoromycota, Olpidiomycota, Sanchytriomycota, and Zoopagomycota. In the TBS group, the relative abundance of Ascomycota and Chytridiomycota increased by 5.07%. At the same time, Mucoromycota, Basidiomycota, Olpidiomycota, Blastocladiomycota, and Ascomycota decreased by 15.06%, 0.48%, 38.36%, and 21.25%, respectively, compared to the healthy group (**Figure 3C**). At the fungal genus level, the top 10 taxa included *Fusarium*, *Mortierella*, *Alternaria*, *Chaetomium*, *Botryotrichum*, *Olpidium*, *Plectosphaerella*, *Penicillium*, *Humicola*, and *Linnemannia*. In the TBS group, the abundances of *Mortierella*, *Chaetomium*, *Humicola*, *Olpidium*, *Penicillium*, and *Linnemannia* decreased by 10.63%, 6.03%, 2.02%, 38.36%, 39.32%, and 40.37%, respectively, while *Fusarium*, *Alternaria*, *Botryotrichum*, and *Plectosphaerella* increased by 7.42%, 23.02%, 31.62%, and 74.03%, respectively, compared to the healthy group (**Figure 3D**).

An analysis of microorganisms with potential biocontrol functions and phytopathogenic fungal communities further revealed distinct microbial distributions between healthy and TBS-infected rhizospheres. Specifically, the healthy tobacco rhizosphere was significantly enriched with beneficial microbes, such as *Pseudomonas*, *Paenibacillus*, and *Penicillium*, whose abundances were 3.64%, 9.23%, and 64.79% higher, respectively, than those in the TBS group (**Figure 3E**). In contrast, the TBS-infected rhizosphere exhibited a greater accumulation of pathogenic fungi, such as *Fusarium*, *Puccinia*, and *Rhizoctonia*, with abundances 7.42%, 61.46%, and 21.85% higher, respectively, compared with the healthy group (**Figure 3F**). Collectively, these results suggest that TBS occurrence may be closely associated with shifts in rhizosphere microbial composition.

### 3.4 Biomarkers of the healthy and TBS-infected tobacco rhizosphere microbes

**Figure 4:**
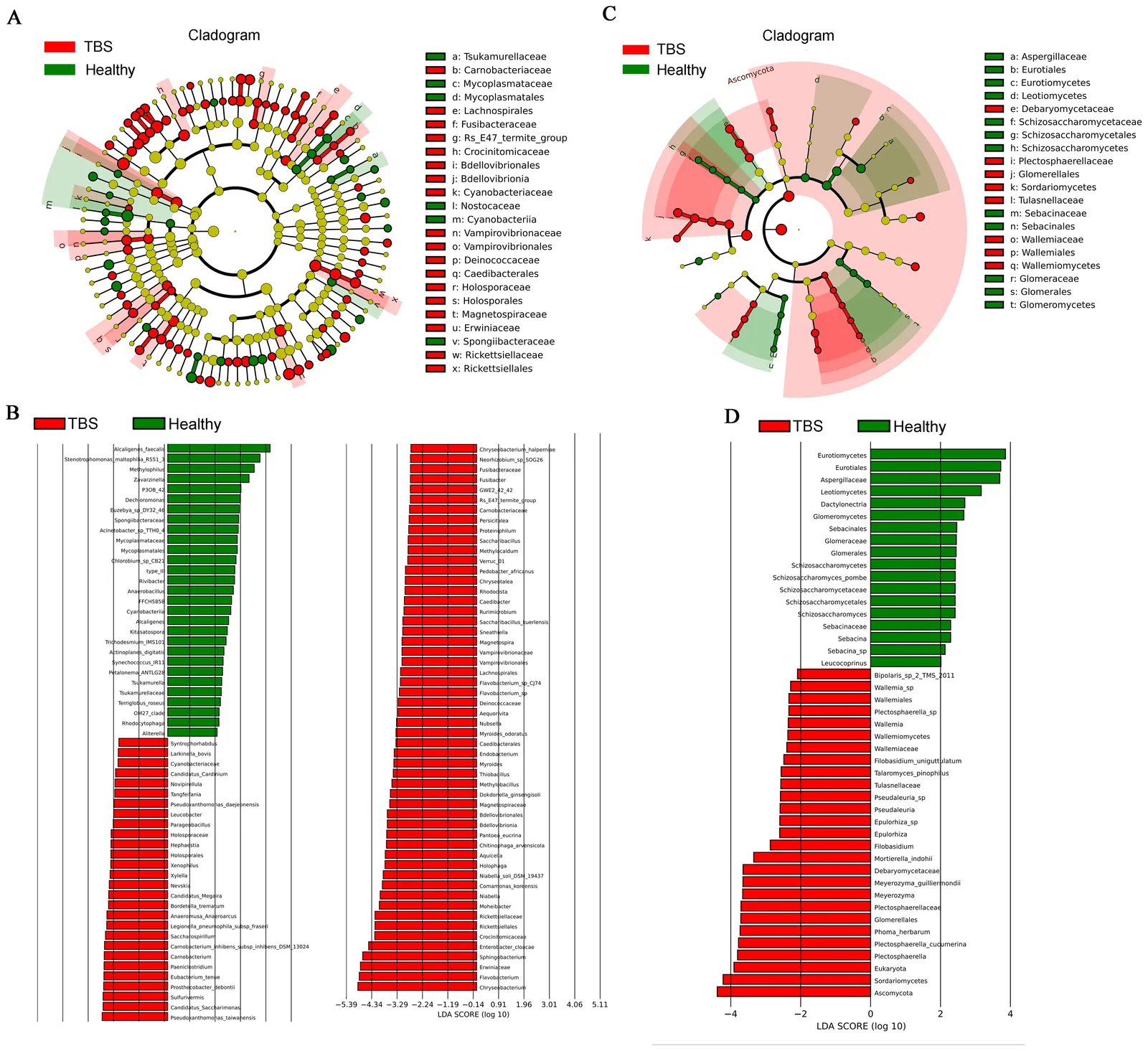
Identification of potential biomarkers distinguishing healthy and TBS states. Evolutionary branch diagram of bacteria **(A)** and fungi **(B)**. LDA value distribution histogram of bacteria **(C)** and fungi **(D)**.

LEfSe analysis was conducted on tobacco rhizosphere bacteria exhibiting significant differences in abundance. Across healthy and TBS-infected soils, a total of 46 phyla, 103 classes, 236 orders, 447 families, 1,720 genera, and 825 species were identified as bacterial biomarkers. Using LDA > 2 and *P* < 0.05 as selection criteria, 83 bacterial taxa were identified as biomarkers of TBS-infected tobacco, with representative taxa including *Chryseobacterium*, *Flavobacterium*, *Erwiniaceae*, etc. Conversely, 30 bacterial taxa were characterized as biomarkers of healthy soils, comprising representative taxa such as *Nostocaceae*, *Alcaligenes faecalis*, and *Stenotrophomonas maltophilia* R551_3 (**Figures 4A, B**).

Similarly, LEfSe analysis of tobacco rhizosphere fungi revealed significant differences in abundance between healthy and TBS-infected soils. A total of 10 phyla, 49 classes, 157 orders, 441 families, 1,415 genera, and 3,416 species of fungal biomarkers were detected. With LDA > 2 and *P* < 0.05 as thresholds, 27 fungal taxa served as biomarkers for TBS-infected tobacco, with key representatives including *Ascomycota*, *Sordariomycetes*, and *Eukaryota*. In contrast, 18 fungal taxa were identified as biomarkers for healthy tobacco, with key representatives including *Eurotiomycetes*, *Eurotiales*, *Aspergillaceae*, etc. (**Figures 4C, D**).

### 3.5 Microbial functional analysis and co-occurrence network of healthy and TBS-infected tobacco rhizosphere microbes

**Figure 5:**
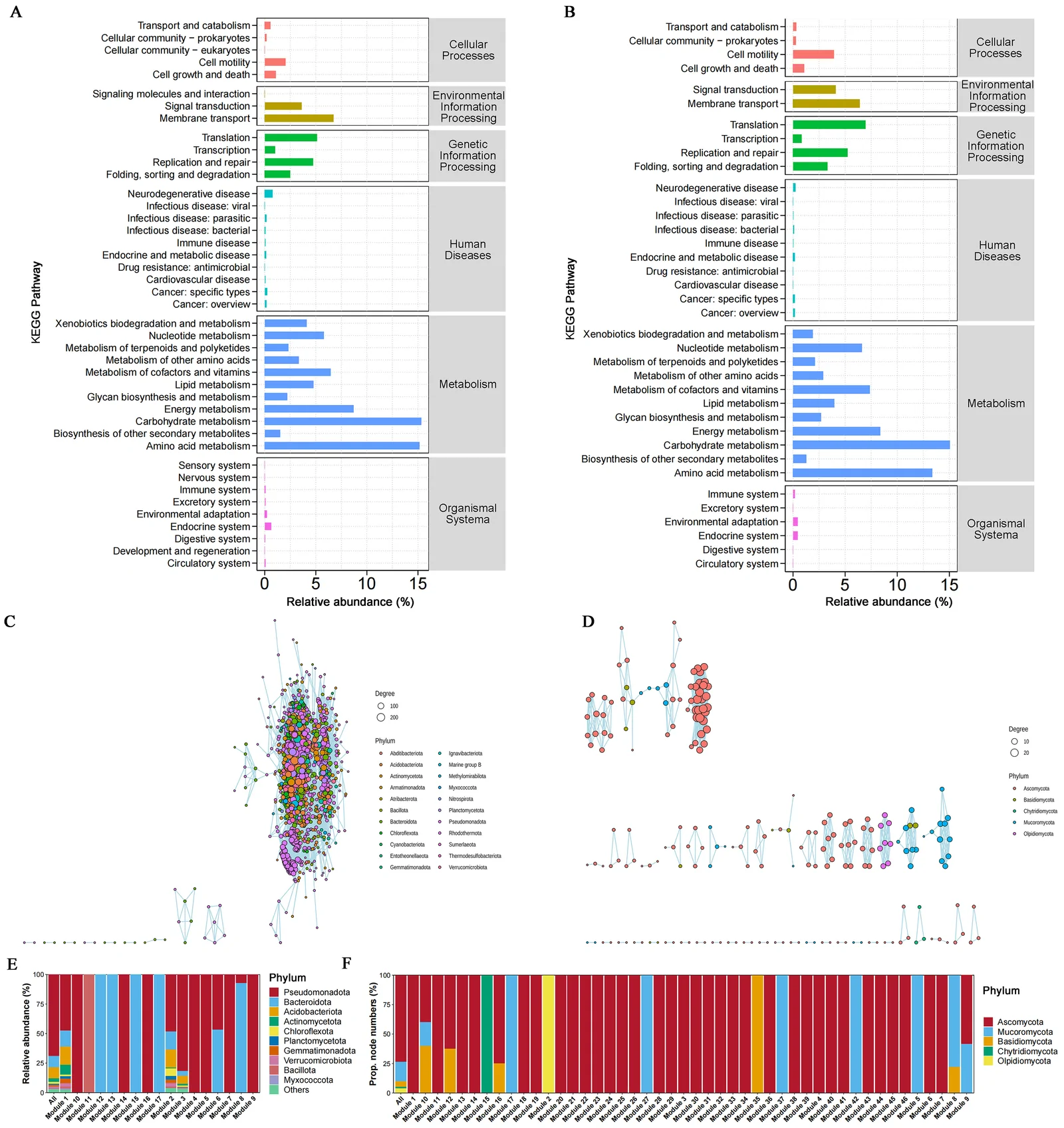
KEGG pathway analysis of differences between bacteria **(A)** and fungi **(B)** in healthy and TBS rhizosphere soil. Co-occurrence network of bacteria **(C, E)** and fungi **(D, F)** in healthy and TBS rhizosphere soil. KEGG pathway analysis of differences between bacteria **(E)** and fungi **(F)** in healthy and TBS rhizosphere soil.

PICRUSt was used to predict the functional potential of healthy and TBS-infected tobacco rhizosphere microbes. The weighted NSTI values ranged from 0.026 to 0.182, with a mean of 0.116 ± 0.031 (median = 0.120). The results indicated that both the rhizosphere bacterial (**Figure 5A**) and fungal (**Figure 5B**) communities exhibited highly similar pathway types, primarily involving six KEGG pathways: metabolism, genetic information processing, environmental information processing, cellular processes, organismal systems, and human diseases. However, differences were observed in the intensity of specific functions. Specifically, Bacteria exhibited stronger amino acid metabolism, transport, and catabolism, whereas fungi showed enhanced nucleotide metabolism and cell motility. These predicted functional differences and complementarities suggest a potential division of labor and cooperative interactions between bacteria and fungi within the rhizosphere microbiome, which may promote the stable coexistence of microbial communities and potentially provide more comprehensive ecological services for the host plant. Although the relatively low NSTI values indicate a reasonable level of prediction accuracy, these functional profiles were inferred using PICRUSt from marker gene sequences rather than being directly measured; therefore, they may represent potential functions only and should be interpreted with caution.

Co-occurrence network analysis was performed to explore microbial co-occurrence patterns and to identify differences between sampling groups. The results found that the bacterial co-occurrence network in both healthy and TBS rhizosphere soils was divided into 40 modules, with strong inter-module interactions (**Figure 5C**). Specifically, modules 10, 14, 16, 4, 5, 7, and 9 contained only Pseudomonadota, whereas modules 12, 13, 15, and 17 consisted exclusively of Bacteroidota. Modules 6 and 8 included bacteria from both phyla, while module 11 comprised only Verrucomicrobiota. Modules 1, 2, and 3 exhibited the highest bacterial diversity, encompassing Pseudomonadota, Bacteroidota, Acidobacteriota, Actinomycetota, and Chloroflexota (**Figure 5D**). The fungal co-occurrence network in the healthy and TBS rhizosphere soils was divided into 47 modules, with weaker interconnections compared to the bacterial network (**Figure 5E**). Among these, 33 modules—such as modules 1, 10, 13, 14, 18, and 19—contained only Ascomycota. Modules 5, 17, 27, 37, and 42 consisted solely of Mucoromycota, while module 15 contained only Chytridiomycota, and module 2 included only Olpidiomycota. Module 35 was composed exclusively of Basidiomycota. Modules 12 and 16 contained both Ascomycota and Basidiomycota, whereas module 8 included Mucoromycota and Basidiomycota. Module 10 encompassed Ascomycota, Basidiomycota, and Mucoromycota (**Figure 5F**).

These results indicate that the tobacco rhizosphere microbial community exhibits a “bacteria-dominated modular interaction with highly phylum-segregated fungi” pattern. Such an imbalance in cross-domain microbial interactions may represent a critical factor contributing to the development of TBS.

### 3.6 Influences of environmental factors on rhizosphere microbiota

**Figure 6:**
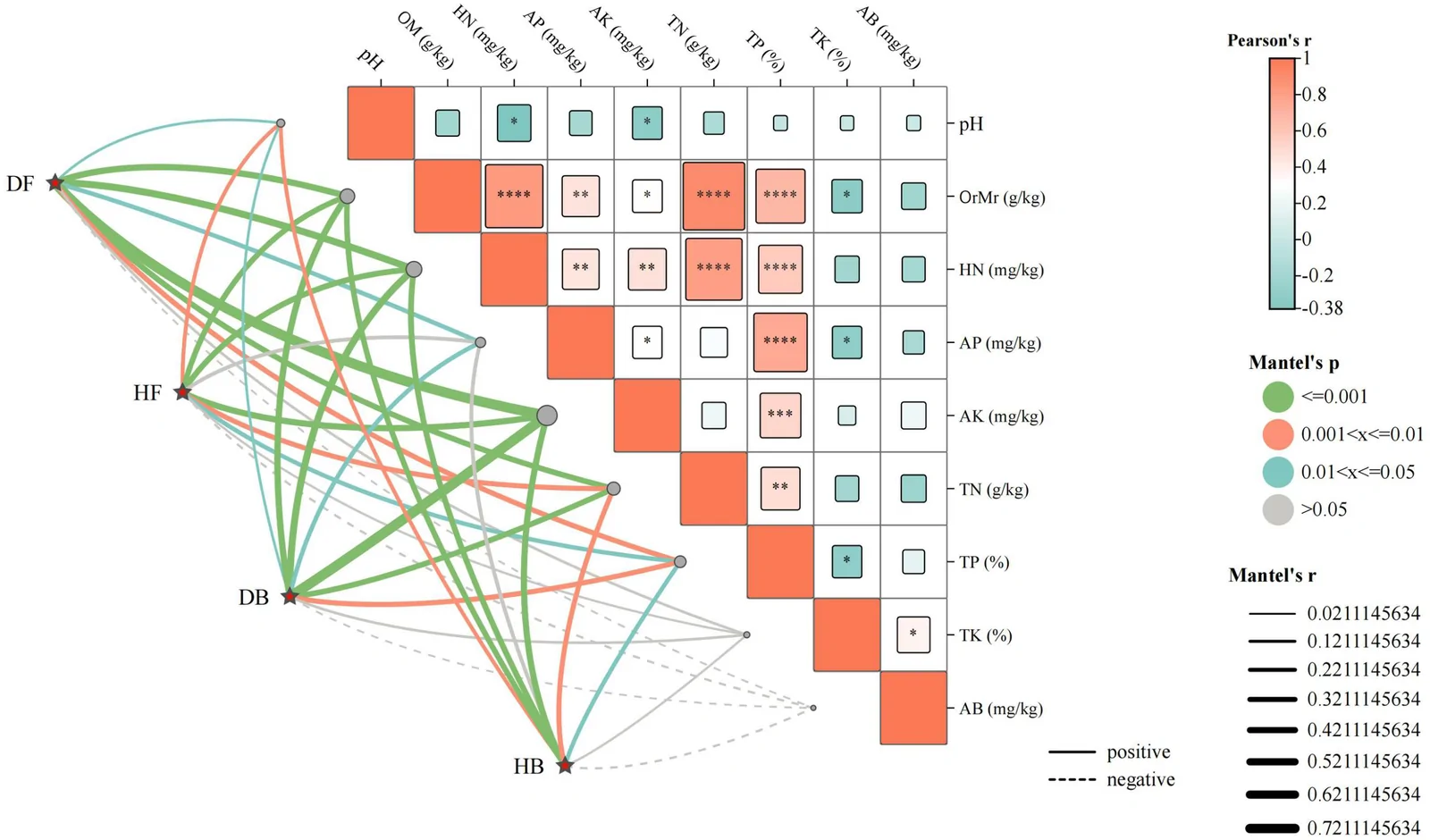
Correlation analysis between soil properties and microbial communities. OM, Organic matter; HN, Hydrolyzable nitrogen; AP, Available phosphorus; AK, Available potassium; TN, Total Nitrogen; TP, Total phosphorus; TK, Total potassium; AB, Available boron. DF, Rhizosphere fungal abundance of diseased tobacco; HF, Rhizosphere fungal abundance of healthy tobacco; DB, rhizosphere bacterial abundance of diseased tobacco; HB, Rhizosphere bacterial abundance of healthy tobacco.

To investigate the relationships between soil properties and rhizosphere microbial communities, we analyzed key soil physicochemical parameters. As shown in **Table 1**, no significant differences were observed in pH, OM, HN, AP, or AK between healthy and diseased soils (all *P* > 0.05). To further assess the associations between soil properties and microbial community assembly, correlation analyses were performed. The results showed that the community structures of both rhizosphere bacteria and fungi were significantly and positively correlated with soil organic matter, hydrolyzable nitrogen, and available potassium in both healthy and diseased tobacco plants (**Figure 6**). Taken together, these findings suggest that the rhizosphere microbiome may help maintain soil fertility and preserve core functional stability under pathogen pressure.

**Table 1:** Physicochemical properties of soil in TBS and healthy group.

Group	pH	OM (g/kg)	HN (mg/kg)	AP (mg/kg)	AK(mg/kg)	TN(g/kg)	TP (%)	TK (%)	AB (mg/kg)
Healthy	6.62±1.02	33.11±19.70	238.15±162.59	138.62±106.36	993.67±656.64	2.01±1.17	0.12±0.06	1.80±0.57	65.98±22.39
TBS	6.55±0.99	33.12±17.58	246.00±135.12	145.49±126.67	1077.51±657.83	2.02±1.01	0.12±0.05	1.84±0.60	67.12±22.85

OM: Organic Matter; HN: Hydrolyzable nitrogen; AP: Available phosphorus; AK: Available potassium; TN: Total Nitrogen; TP: Total phosphorus; TK: Total potassium; AB: Available boron.

Furthermore, redundancy analysis (RDA) was conducted to evaluate the effects of tobacco cultivar and elevation on rhizosphere microbial community composition. The results showed that neither cultivar nor elevation significantly explained the overall variation in bacterial (*P* = 0.949) or fungal (*P* = 0.334) communities (**Supplementary Figure 1**). Therefore, the differences in rhizosphere microbial communities between healthy and diseased plants were unlikely to be driven by cultivar or elevation, thereby further supporting the conclusion that the observed community shifts were primarily associated with TBS occurrence.

### 3.7 Isolation of culturable rhizosphere microbes and screening of *P. nicotianae* antagonistic strains

High-throughput sequencing results suggested that the rhizosphere soil of both healthy and TBS-infected tobacco plants harbors biocontrol microorganisms that may influence plant metabolic processes and environmental adaptation. To further explore these findings and obtain functional microbial resources, we isolated and cultured cultivable microorganisms from the same soil samples. A total of 204 bacterial strains (**Supplementary Figure 2A**) and 95 fungal strains (**Supplementary Figure 2B**) were isolated.

**Figure 7:**
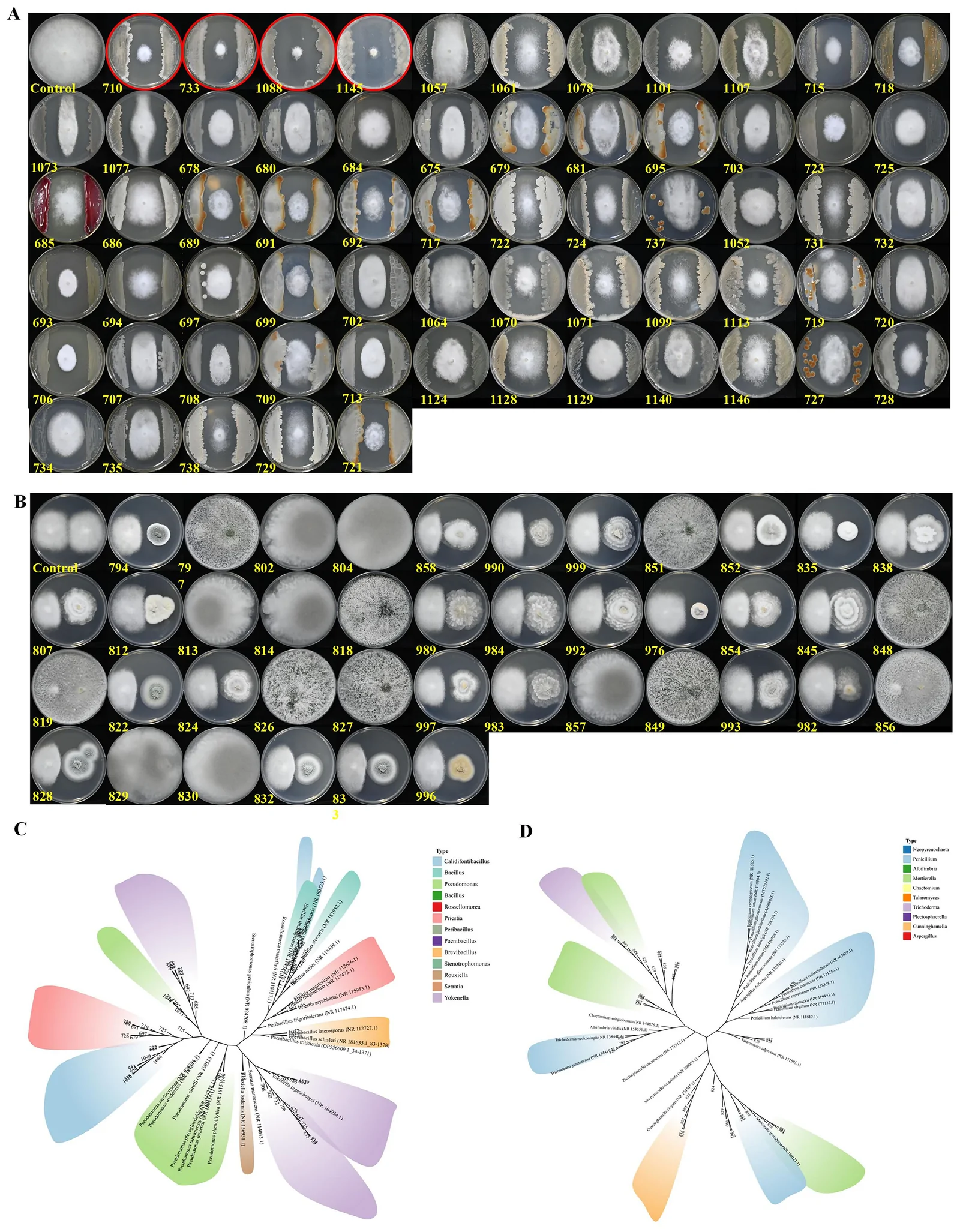
Selection of *P. nicotianae* antagonistic strains. **(A)** *P. nicotianae* antagonistic bacteria; **(B)** *P. nicotianae* antagonistic fungi; **(C)** Phylogenetic analysis of *P. nicotianae* antagonistic bacteria; **(D)** Phylogenetic analysis of *P. nicotianae* antagonistic fungi.

Subsequently, antagonistic strains against *P. nicotianae* were screened using a dual-culture assay. A total of 64 antagonistic bacterial strains (**Figure 7A**) and 41 antagonistic fungal strains (**Figure 7B**) were obtained. Among them, bacterial strains 710, 733, 1088, and 1145 exhibited particularly strong inhibition against *P. nicotianae*. To identify their taxonomic classification, 16S rDNA and ITS sequencing were performed on all 105 antagonistic strains. The results revealed that the 64 antagonistic bacterial strains were primarily classified into *Yokenella* (25.0%), *Priestia* (17.0%), *Pseudomonas* (15.9%), and *Calidifontibacillus* (15.9%) (**Figure 7C**). The 41 fungal strains were primarily classified into *Penicillium* (27.1%), *Mortierella* (24.2%), and *Trichoderma* (10.0%) (**Figure 7D**). Detailed identification results for all isolated strains are provided in **Supplementary Table 2**.

### 3.8 Antagonistic effects of strains 710, 733, 1088, and 1145 on *P. nicotianae*

**Figure 8:**
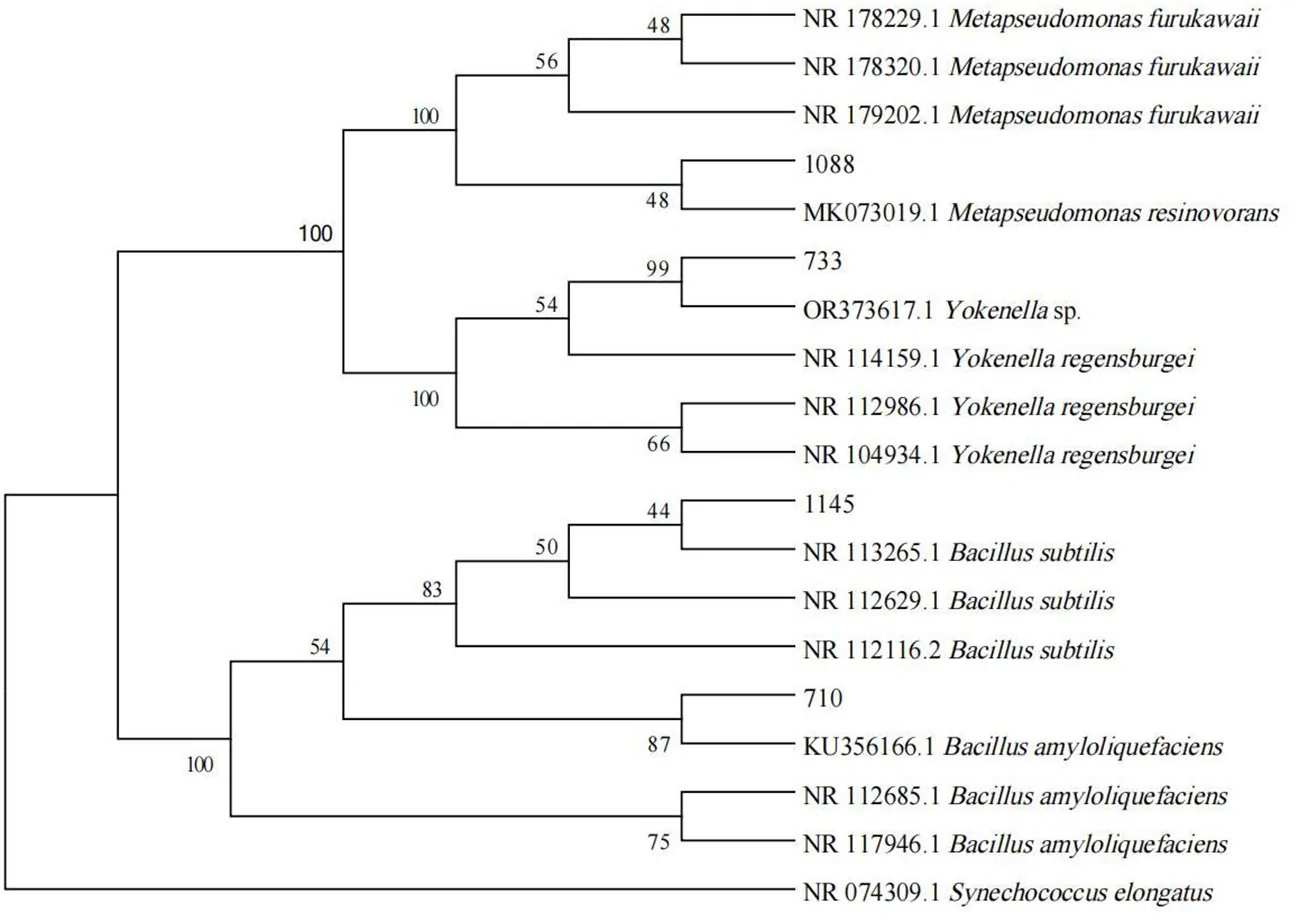
Phylogenetic analysis of strain 710, 733, 1088 and 1145.

**Figure 9:**
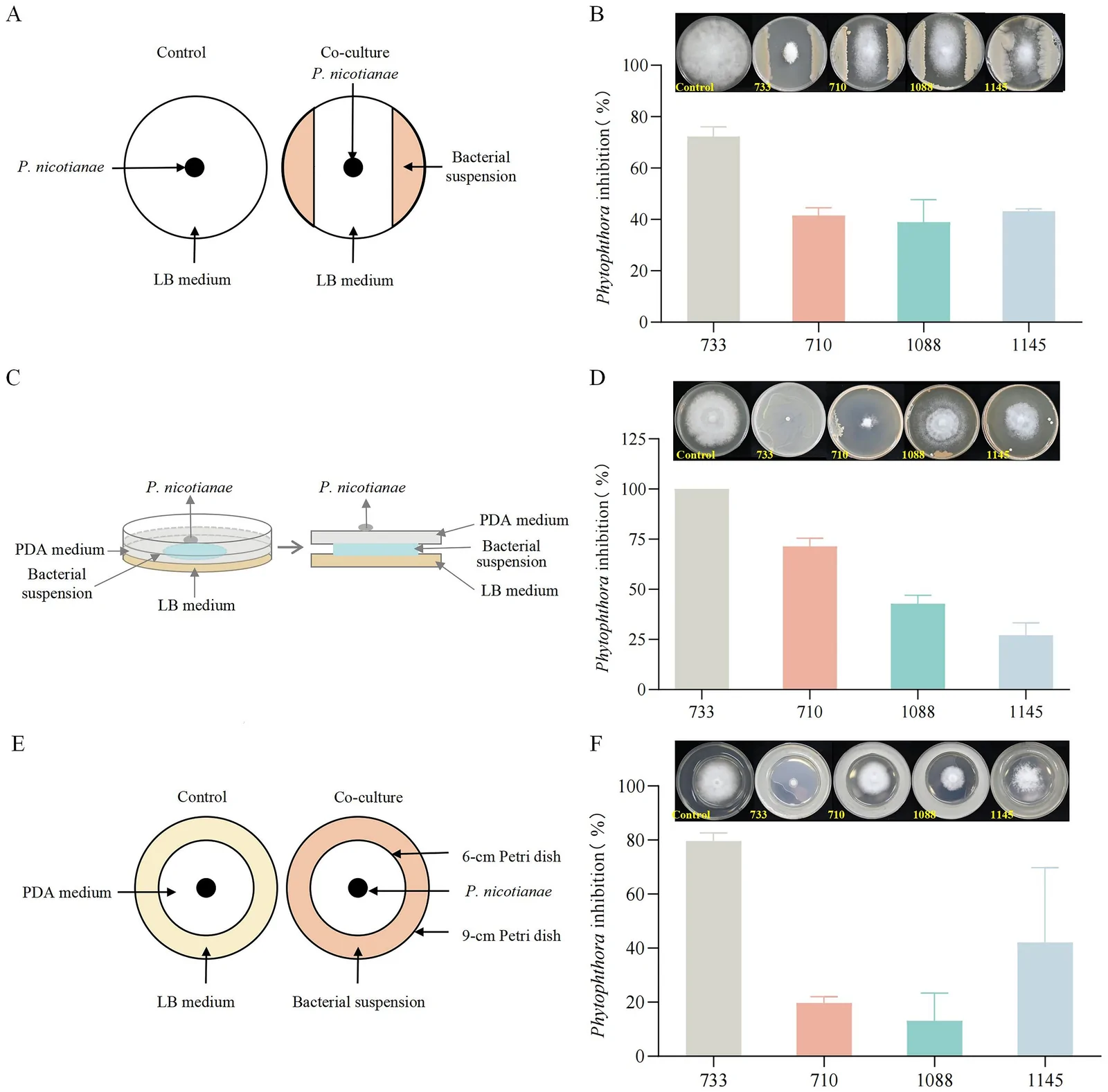
The inhibitory effects of strains 710, 733, 1088 and 1145 on *P. nicotianae*. The mode diagrams **(A)** and plate cultures **(B)** for the confrontation tests; The mode diagrams **(C)** and plate cultures **(D)** for the sandwich agar plate; The mode diagrams **(E)** and plate cultures **(F)** for the donut plate method.

Taxonomic classification based on 16S rDNA sequencing confirmed that strain 710 was *Bacillus amyloliquefaciens*, strain 733 was *Yokenella* sp., strain 1088 was *Metapseudomonas resinovorans*, and strain 1145 was *Bacillus subtilis* (**Figure 8**). To further evaluate the biocontrol potential of the isolates 710, 733, 1088, and 1145, the inhibitory effects were systematically validated using the confrontation plate, sandwich agar plate, and double plate methods. Strain 733 exhibited the most potent inhibitory activity among the tested isolates. Specifically, the confrontation plate assays revealed that strains 733, 710, 1088, and 1145 inhibited the mycelial growth of *P. nicotianae* by 72.29%, 39.96%, 38.96%, and 43.17%, respectively (**Figures 9A, B**). In the sandwich agar plate test, the inhibition rates of the soluble metabolites of these strains against *P. nicotianae* were 100%, 71.46%, 42.92%, and 27.08%, respectively (**Figures 9C, D**). Additionally, the volatile metabolites produced by these strains, as measured by the donut plate method, suppressed *P. nicotianae* by 79.61%, 19.74%, 13.16%, and 42.11%, respectively (**Figures 9E, F**).

## 4 Discussion

In this study, high-throughput sequencing was employed to systematically compare the composition, diversity, and functional profiles of bacterial and fungal communities in the rhizosphere soils of healthy and TBS-susceptible tobacco plants. Furthermore, strains exhibiting antagonistic activity against *P. nicotianae* were isolated and screened. Overall, both healthy and diseased samples exhibited highly complex bacterial and fungal communities, with no significant differences observed in the α- and β-diversity between the two groups (**Figures 2A–D**), consistent with previous reports (Ma et al., 2023). However, contrasting findings have been reported in other studies. For instance, Tang et al. found that α-diversity indices of rhizosphere microorganisms in the TBS group were significantly lower compared to the healthy group (Tang et al., 2023). Similarly, resistant tobacco varieties were able to assemble distinct microbial communities with higher network complexity and diversity (Yang et al., 2024). These discrepancies may stem from the specific timing of sampling during disease progression.

Research suggests that microbial communities may remain relatively stable during the early infection stages, primarily owing to limited pathogen colonization and the lack of strong host defense activation (Wei et al., 2019). However, as disease progresses, environmental stress and altered plant physiology may substantially restructure these communities (Shou-ming et al., 2023). During late disease development, certain beneficial taxa may recolonize the rhizosphere and partially restore ecological functions (Fan et al., 2023; Zhang Y et al., 2024). In this study, rhizosphere soil was collected at disease grade 7, when the microbiome may have already partially recovered. This possibility may explain the absence of significant differences in alpha and beta diversity. Therefore, future studies should incorporate time-series sampling to clarify temporal changes and interactions between TBS development and the rhizosphere microbiome.

Although the overall microbial diversity did not differ significantly, the community composition exhibited pronounced shifts in specific taxa. Previous studies have indicated that disease occurrence is often associated with a decrease in the abundance of beneficial rhizosphere microbes and an increase in harmful microbes. For example, Tang et al. (Tang et al., 2023) reported that the healthy tobacco group was enriched with beneficial rhizosphere microorganisms, such as *Streptomyces* and *Amycolatopsis*. However, the abundance of these beneficial microbes decreased, and the microbial interaction network was weakened after infection with TBS. Similarly, Yang et al. (Yang et al., 2025) observed that the relative abundance of harmful microbes, such as *Alternaria*, *Fusarium*, and *Filobasidium*, significantly increased in tobacco plants infected with Fusarium root rot, while the relative abundance of beneficial microbes, including *Lysobacter*, *Streptomyces*, *Mortierella*, and *Penicillium*, significantly decreased. In another study, Li et al. (Li B, et al., 2023) found that the rhizosphere soil of the TBS-resistant tobacco variety (HB202) was significantly enriched with beneficial bacteria, such as *Bryobacter*, *Chthoniobacter*, and *Ferruginibacter*. However, the rhizosphere of the TBS-susceptible tobacco variety (XY3) was enriched with harmful fungi, such as *Fusarium* and *Pseudotricharina.* Consistent with these findings, beneficial microorganisms such as *Pseudomonas*, *Paenibacillus*, and *Penicillium* were enriched in the rhizosphere of healthy tobacco plants (**Figure 3E**), whereas harmful microorganisms, such as *Fusarium*, *Puccinia*, and *Rhizoctonia*, were more abundant in TBS-affected soils (**Figure 3F**). These results further support the notion that the occurrence of TBS drives a shift in the rhizosphere microbial community from a healthy state toward a pathogen-dominated, imbalanced condition.

Notably, no OTUs affiliated with *Phytophthora* were detected in this study, which is consistent with previous reports (Xiang et al., 2019; Cheng et al., 2024). This absence likely reflects primer bias. Specifically, universal ITS primers were used, which are primarily designed for fungi, whereas *P. nicotianae* belongs to the Oomycota. Therefore, these primers are inefficient for amplifying Oomycete DNA (Legeay et al., 2019; Tremblay, 2019), making the non-detection of Phytophthora unsurprising.

The rhizosphere soil is rich in disease-suppressive microorganisms. In recent years, numerous studies have isolated a variety of rhizosphere microorganisms exhibiting antagonistic activity against *P. nicotianae*. Among these microorganisms, *Bacillus* has been widely reported for its biocontrol potential against *P. nicotianae*. For example, Zeng et al. (Zeng et al., 2023) found that *Bacillus amyloliquefaciens* CAS02 disrupted the mycelial structure of *P. nicotianae*, inhibiting its growth, reducing disease indices, and improving plant height and maximum leaf area of tobacco seedlings. Similarly, Zhang et al. (Zhang et al., 2026) demonstrated that *Bacillus subtilis* not only promoted tobacco growth but also significantly reduced TBS incidence, achieving a control efficacy of 53.45%. Ding et al. (Ding et al., 2021) conducted dual-culture tests *in vitro* and showed that *Bacillus velezensis* inhibited the growth of *P. nicotianae* and exhibited over 70% control efficiency against TBS in field experiments. These studies strongly support the great potential of *Bacillus* sp. in biocontrol of TBS. Consistent with these previous findings, the *Bacillus amyloliquefaciens* (710) and *Bacillus subtilis* (1145) strains isolated in this study also exhibited strong inhibitory effects against *P. nicotianae* (**Figures 8A, B**), further reinforcing the evidence of Bacillus species as valuable biocontrol agents.

Moreover, previous studies have demonstrated that *Yokenella* sp. possesses both plant growth-promoting and antimicrobial potential. For example, inoculation with *Yokenella regensburgei* improved soil quality and promoted plant growth (Li et al., 2026). Furthermore, an endophytic bacterium of the genus *Yokenella*, isolated from the roots of vetiver (*Chrysopogon zizanioides* (L.) Roberty), together with its lipopeptide metabolites (surfactins and plipastatins), showed antifungal activity against F*usarium graminearum* (Munakata et al., 2022). Similarly, *Metapseudomonas* sp. also exhibits biocontrol potential. For instance, *Metapseudomonas* sp. strain CR1201T, isolated from citrus rhizosphere soil, produced indole-3-acetic acid and siderophores and exhibited strong antagonistic activity against *Xanthomonas citri subsp*. citri (Yang et al., 2026). However, the inhibitory effects of *Yokenella* sp. and *Metapseudomonas* sp. against TBS and its causal pathogen, *P. nicotianae*, have not yet been reported. To the best of current knowledge, this is the first report demonstrating that these two genera exhibit direct antagonistic activity against *P. nicotianae*, thereby suggesting that their antimicrobial secondary metabolites may have a broader inhibitory spectrum than previously recognized.

Microorganisms can inhibit the growth of pathogenic microbes by producing soluble and volatile antimicrobial compounds. For instance, Guo et al. (Guo et al., 2020) found that the proteins/peptides in the extracellular metabolites of *Bacillus velezensis* can inhibit the growth of *P. nicotianae* by irreversibly damaging its cell wall and membrane. Similarly, Meng et al. (Meng et al., 2024) discovered that the lipopeptide active substances produced by the B4-7 strain (*Bacillus velezensis*) exhibited strong antibacterial activity. Raio et al. (Raio et al., 2023) demonstrated that *Stenotrophomonas rhizophila* Ep2.2 inhibits the growth of *Botrytis cinerea* through emitting volatile organic compounds, limiting leaf infection and priming plant defense genes. In this study, strains 710, 733, 1088, and 1145 produced both soluble (**Figures 8C, D**) and volatile metabolites (**Figures 8E, F**) that significantly inhibited *P. nicotianae* growth. Notably, strain 733 showed the strongest antibacterial activity, with its soluble and volatile metabolites inhibiting *P. nicotianae* mycelial growth by 100% and 79.61%, respectively. These findings provide a solid foundation for the potential use of *Yokenella* sp.733 in the biocontrol of *P. nicotianae*.

## 5 Conclusion

In summary, this study combined high-throughput sequencing with strain isolation and antagonistic screening to characterize the associations between tobacco rhizosphere microbiota and TBS and to identify candidate biocontrol strains. Although no significant differences in alpha or beta diversity were detected between healthy and diseased rhizospheres, specific microbial taxa showed distinct enrichment patterns. Healthy rhizospheres were enriched in potentially beneficial bacteria, including *Pseudomonas* and *Paenibacillus*, whereas diseased rhizospheres contained higher abundances of potentially plant-pathogenic fungi, including *Fusarium* and *Alternaria*. Among 105 antagonistic isolates, *Yokenella* sp. 733 and *Metapseudomonas resinovorans* 1088 inhibited *P. nicotianae in vitro*. Strain 733 showed the strongest antagonistic activity, with its living cells, soluble metabolites, and volatile metabolites producing inhibition rates of 72.29%, 100%, and 79.61%, respectively. These findings broaden the microbial resources available for TBS control and identify strain 733 as a candidate for further evaluation.

Several limitations should be acknowledged. Field management practices were not systematically recorded, preventing assessment of their contributions to rhizosphere microbial variation. Moreover, the antagonistic effects of the isolated strains were demonstrated only *in vitro*, while their active metabolites and underlying mechanisms remain unresolved. Future studies should therefore validate the efficacy and stability of these strains in pot and field trials, incorporate agronomic variables into multivariate analyses, and identify the metabolites and mechanisms responsible for their antagonistic activity.

## Data Availability

The data presented in the study are deposited in the NCBI Sequence Read Archive (SRA) under BioProject accession number PRJNA1509760.

## Supplementary material

Supplementary Figure 1 RDA analysis reveals the effects of altitude and tobacco cultivar on the rhizosphere microbial communities of healthy and TBS-affected tobacco plants. **(A)** Effects of altitude and tobacco cultivar on rhizosphere bacterial communities; **(B)** Effects of altitude and tobacco cultivar on rhizosphere fungal communities.

Supplementary Figure 2 Isolation of rhizosphere microbes. Bacterial **(A)** and fungal **(B)** strains were isolated from rhizosphere soil samples.
