# Supplementary material for: Rhizosphere microbial diversity analysis reveals enrichment of biocontrol taxa in healthy tobacco plants and yields novel antagonists against Phytophthora nicotianae
Source: Front Plant Sci. 2026 Sep 7;17:1889242. doi: 10.3389/fpls.2026.1889242 (PMC13590766; doi:10.3389/fpls.2026.1889242)
Supplement: Supplementary file 1 [file Table1.doc]

**Supplementary Table 1 Information table of rhizosphere soil samples of healthy and TBS**

| **NO.** | **Sample Name** | **Health/TBS** | **Quantity** | **Sampling location** | **latitude and longitude** | **altitude（m）** | **Collection Time** | **Variety** |
| --- | --- | --- | --- | --- | --- | --- | --- | --- |
| 1 | XYC80-82 | TBS | 3 | Xiaolinyi Village, Wanqiao Town, Dali City | 100°8′30′′ E, 25°47′17′′ N | 1933 | June 24, 2024 | K326 |
| 2 | XYC83-85 | Health | 3 |  |  |  |  |  |
| 3 | XYC86-88 | TBS | 3 | Sutun Village, Bonan Town, Yongping County | 99°32′13′′ E, 25°27′0′′ N | 1610 | June 25, 2024 | K326 |
| 4 | XYC89-91 | Health | 3 |  |  |  |  |  |
| 5 | XYC82-94 | TBS | 3 | Shijia Village, Longmen Township, Yongping County | 99°30′55′′ E, 25°30′49′′ N | 1692 | June 25, 2024 | K326 |
| 6 | XYC95-97 | Health | 3 |  |  |  |  |  |
| 7 | XYC98-100 | TBS | 3 | Jiluhe Village, Longtan Town, Yangbi County | 99°51′42′′ E, 25°21′6′′ N | 1525 | June 26, 2024 | K326 |
| 8 | XYC101-103 | Health | 3 |  |  |  |  |  |
| 9 | XYC104-106 | TBS | 3 | Caibai Village, Jijie Town, Yangbi County | 99°55′20′′ E, 25°16′1′′ N | 1525 | June 26, 2024 | K326 |
| 10 | XYC107-109 | Health | 3 |  |  |  |  |  |
| 11 | XYC110-112 | TBS | 3 | Dapai Village, Shangguan Town, Dali City | 100°12′43′′ E, 25°95′19′′ N | 1971 | June 26, 2024 | Yunyan 87 |
| 12 | XYC113-115 | Health | 3 |  |  |  |  |  |
| 13 | XYC116-118 | TBS | 3 | Xinze Village, Hedian Town, Xiangyun County | 100°43′36′′ E, 25°35′26′′ N | 1952 | June 27, 2024 | Yunyan 99 |
| 14 | XYC119-121 | Health | 3 |  |  |  |  |  |
| 15 | XYC122-124 | TBS | 3 | Shadi Village, Liuchang Town, Xiangyun County | 100°46′3′′ E, 25°31′22′′ N | 1984 | June 27, 2024 | Yunyan 99 |
| 16 | XYC125-127 | Health | 3 |  |  |  |  |  |
| 17 | XYC128-130 | TBS | 3 | Jiyi Village, Xiyi Town, Heqing County | 100°10′14′′ E, 26°16′18′′ N | 2351 | June 28, 2024 | Yunyan 99 |
| 18 | XYC131-133 | Health | 3 |  |  |  |  |  |
| 19 | XYC134-136 | TBS | 3 | Pineapple Village, Songgui Town, Heqing County | 100°11′40′′ E, 26°22′34′′ N | 1983 | June 28, 2024 | Yunyan 99 |
| 20 | XYC137-139 | Health | 3 |  |  |  |  |  |
| 21 | XYC140-142 | TBS | 3 | Shangang Village, Zhoucheng Town, Binchuan County | 100°31′45′′ E, 25°38′23′′ N | 1587 | June 29, 2024 | Yunyan 99 |
| 22 | XYC143-145 | Health | 3 |  |  |  |  |  |
| 23 | XYC146-148 | TBS | 3 | XiongLume Village, Qiaodian Town, Binchuan County | 100°36′53′′ E, 25°39′13′′ N | 1861 | June 29, 2024 | Yunyan 99 |
| 24 | XYC149-151 | Health | 3 |  |  |  |  |  |
| 25 | XYC152-154 | TBS | 3 | Jianshe Village, Weibaoshan Township, Weishan County | 100°19′5′′ E, 25°10′45′′ N | 1625 | June 29, 2024 | Honghua Dajinyuan |
| 26 | XYC155-157 | Health | 3 |  |  |  |  |  |
| 27 | XYC158-160 | TBS | 3 | Xianjue Village, Nanzhao Town, Weishan County | 100°18′23′′ E, 25°12′34′′ N | 1674 | June 25, 2024 | Honghua Dajinyuan |
| 28 | XYC161-163 | Health | 3 |  |  |  |  |  |
| 29 | XYC164-166 | TBS | 3 | Zuobei Village, Miaojie Town, Weishan County | 100°15′25′′ E, 25°20′35′′ N | 1699 | June 25, 2024 | Honghua Dajinyuan |
| 30 | XYC167-169 | Health | 3 |  |  |  |  |  |
| 31 | XYC170-172 | TBS | 3 | Dingjiazhuang, Dacang Town, Weishan County | 100°14′49′′ E, 25°21′6′′ N | 1696 | June 25, 2024 | Honghua Dajinyuan |
| 32 | XYC173-175 | Health | 3 |  |  |  |  |  |
| 33 | XYC176-178 | TBS | 3 | Zhangbao Village, Yongjian Town, Weishan County | 100°11′23′′ E, 25°25′28′′ N | 1722 | June 25, 2024 | Honghua Dajinyuan |
| 34 | XYC179-181 | Health | 3 |  |  |  |  |  |
| 35 | XYC182-184 | TBS | 3 | Wenqi Village, Nanjian Town, Nanjian County | 100°35′55′′ E, 24°59′59′′ N | 2032 | June 27, 2024 | Honghua Dajinyuan |
| 36 | XYC185-187 | Health | 3 |  |  |  |  |  |
| 37 | XYC188-190 | TBS | 3 | Dapingdi Community, Yunhua Village Committee, Nanjian Town, Nanjian County | 100°33′57′′ E, 24°60′0′′ N | 2017 | June 27, 2024 | Honghua Dajinyuan |
| 38 | XYC191-193 | Health | 3 |  |  |  |  |  |
| 39 | XYC194-196 | TBS | 3 | Fada Village, Wuliangshan Town, Nanjian County | 100°24′48′′ E, 24°55′30′′ N | 2215 | June 27, 2024 | Honghua Dajinyuan |
| 40 | XYC197-199 | Health | 3 |  |  |  |  |  |
| 41 | XYC200-202 | TBS | 3 | Xiaoboluo Village, Yongcui Township, Nanjian County | 100°32′32′′ E, 24°52′25′′ N | 2219 | June 27, 2024 | Honghua Dajinyuan |
| 42 | XYC203-205 | Health | 3 |  |  |  |  |  |
| 43 | XYC206-208 | TBS | 3 | Akedi Village, Leqiu Township, Nanjian County | 100°21′14′′ E, 25°1′55′′ N | 1641 | June 27, 2024 | Honghua Dajinyuan |
| 44 | XYC209-211 | Health | 3 |  |  |  |  |  |
| 45 | XYC212-214 | TBS | 3 | Xiangyang Village, Deju Township, Midu County | 100°38′26′′ E, 25°6′35′′ N | 1500 | June 28, 2024 | Yunyan 87 |
| 46 | XYC215-217 | Health | 3 |  |  |  |  |  |
| 47 | XYC218-220 | TBS | 3 | Patrol Village, Mizhi Town, Midu County | 100°31′38′′ E, 25°10′12′′ N | 1957 | June 28, 2024 | Yunyan 87 |
| 48 | XYC221-223 | Health | 3 |  |  |  |  |  |
| 49 | XYC224-226 | TBS | 3 | Taojiaying, Xinjie Town, Midu County | 100°27′9′′ E, 25°22′24′′ N | 1661 | June 28, 2024 | Yunyan 87 |
| 50 | XYC227-229 | Health | 3 |  |  |  |  |  |
| 51 | XYC230-232 | TBS | 3 | Zhou Xinying, Xinjie Town, Midu County | 100°25′42′′ E, 25°23′43′′ N | 1675 | June 28, 2024 | Yunyan 87 |
| 52 | XYC233-235 | Health | 3 |  |  |  |  |  |
| 53 | XYC236-238 | TBS | 3 | Dali Wei, Hongyan Town, Midu County | 100°27′0′′ E, 25°23′50′′ N | 1661 | June 28, 2024 | Yunyan 87 |
| 54 | XYC239-241 | Health | 3 |  |  |  |  |  |
| 55 | XYC242-244 | TBS | 3 | Taiping Village, Niujie Township, Eryuan County | 99°59′28′′ E, 26°16′39′′ N | 2085 | June 29, 2024 | Honghua Dajinyuan |
| 56 | XYC245-247 | Health | 3 |  |  |  |  |  |
| 57 | XYC248-250 | TBS | 3 | Guanyin Deng, Qiaohou Township, Eryuan County | 99°45′31′′ E, 26°5′13′′ N | 1785 | June 29, 2024 | Honghua Dajinyuan |
| 58 | XYC251-253 | Health | 3 |  |  |  |  |  |
| 59 | XYC254-256 | TBS | 3 | Tunhu Village, Fengyu Town, Eryuan County | 99°58′7′′ E, 25°57′50′′ N | 1800 | June 29, 2024 | Honghua Dajinyuan |
| 60 | XYC257-259 | Health | 3 |  |  |  |  |  |
| 61 | XYC260-262 | TBS | 3 | New Countryside in Sanying Town, Eryuan County | 99°59′42′′ E, 26°11′23′′ N | 2047 | June 29, 2024 | Honghua Dajinyuan |
| 62 | XYC263-265 | Health | 3 |  |  |  |  |  |
| 63 | XYC266-268 | TBS | 3 | Yonglian Village, Fubi Lake, Eryuan County | 99°58′42′′ E, 26°8′47′′ N | 2028 | June 29, 2024 | Honghua Dajinyuan |
| 64 | XYC269-271 | Health | 3 |  |  |  |  |  |
| 65 | XYC272-274 | TBS | 3 | Jinxing Village, Jinhua Town, Jianchuan County | 99°55′11′′ E, 26°30′4′′ N | 2156 | July 2, 2024 | Honghua Dajinyuan |
| 66 | XYC275-277 | Health | 3 |  |  |  |  |  |
| 67 | XYC278-280 | TBS | 3 | Huilong Village, Diannan Town, Jianchuan County | 99°56′24′′ E, 26°27′12′′ N | 2191 | July 2, 2024 | Honghua Dajinyuan |
| 68 | XYC281-283 | Health | 3 |  |  |  |  |  |
| 69 | XYC284-286 | TBS | 3 | Qiwen Village, Laojunshan Town, Jianchuan County | 99°35′24′′ E, 26°30′21′′ N | 2391 | July 2, 2024 | Honghua Dajinyuan |
| 70 | XYC287-289 | Health | 3 |  |  |  |  |  |
| 71 | XYC290-292 | TBS | 3 | Diansuo Village, Madang Town, Jianchuan County | 99°37′19′′ E, 26°24′20′′ N | 2347 | July 2, 2024 | Honghua Dajinyuan |
| 72 | XYC293-295 | Health | 3 |  |  |  |  |  |
| 73 | XYC296-298 | TBS | 3 | Yanqu Village, Misha Township, Jianchuan County | 99°39′53′′ E, 26°21′55′′ N | 2238 | July 2, 2024 | Honghua Dajinyuan |
| 74 | XYC299-301 | Health | 3 |  |  |  |  |  |
| 75 | XYC302-304 | TBS | 3 | Shuanglong Village, Baishi Town, Yunlong County | 99°26′36′′ E, 26°8′14′′ N | 1886 | July 3, 2024 | K326 |
| 76 | XYC305-307 | Health | 3 |  |  |  |  |  |
| 77 | XYC308-310 | TBS | 3 | Shuanglong Village, Baishi Town, Yunlong County | 99°24′44′′ E, 26°9′8′′ N | 2110 | July 3, 2024 | K326 |
| 78 | XYC311-313 | Health | 3 |  |  |  |  |  |
| 79 | XYC314-316 | TBS | 3 | Yunlong County Mayor Xinxiang Baoluo Village | 99°26′2′′ E, 26°6′18′′ N | 2022 | July 3, 2024 | K326 |
| 80 | XYC317-319 | Health | 3 |  |  |  |  |  |
| 81 | XYC320-322 | TBS | 3 | Yunlong County Mayor Xinxiang Jiaju Village | 99°25′46′′ E, 26°2′24′′ N | 1822 | July 3, 2024 | K326 |
| 82 | XYC323-325 | Health | 3 |  |  |  |  |  |
| 83 | XYC326-328 | TBS | 3 | Yunlong County Mayor Xinxiang Fengsheng Village | 99°25′1′′ E, 25°59′34′′ N | 1929 | July 3, 2024 | K326 |
| 84 | XYC329-331 | Health | 3 |  |  |  |  |  |

**Supplementary Table 2 Identification results of bacteria and fungi that inhibit the growth of *P. nicotianae***

| **Strain No.** | **Species** | **Blast Result** | **Strain No.** | **Species** | **Blast Result** |
| --- | --- | --- | --- | --- | --- |
| 710 | Bacteria | *Bacillus amyloliquefaciens* | 1077 | Bacteria | *Brevibacillus schisleri* |
| 733 | Bacteria | *Yokenella* sp. | 1078 | Bacteria | *Pseudomonas mediterranea* |
| 1088 | Bacteria | *Metapseudomonas resinovorans* | 1099 | Bacteria | *Calidifontibacillus erzurumensis* |
| 1145 | Bacteria | *Bacillus subtilis* | 1101 | Bacteria | *Bacillus sp.* |
| 675 | Bacteria | *Yokenella regensburgei* | 1107 | Bacteria | *Pseudomonas uvaldensis* |
| 678 | Bacteria | *Yokenella regensburgei* | 1113 | Bacteria | *Calidifontibacillus erzurumensis* |
| 679 | Bacteria | *Priestia megaterium* | 1124 | Bacteria | *Bacillus sp.* |
| 680 | Bacteria | *Yokenella regensburgei* | 1128 | Bacteria | *Priestia megaterium* |
| 681 | Bacteria | *Priestia aryabhattai* | 1129 | Bacteria | *Yokenella regensburgei* |
| 684 | Bacteria | *Bacillus aerius* | 1140 | Bacteria | *Pseudomonas phenolilytica* |
| 685 | Bacteria | *Serratia marcescens* | 1146 | Bacteria | *Bacillus amyloliquefaciens* |
| 686 | Bacteria | *Yokenella regensburgei* | 794 | Fungi | *Neopyrenochaeta acicola* |
| 689 | Bacteria | *Yokenella regensburgei* | 797 | Fungi | *Penicillium sp.* |
| 691 | Bacteria | *Priestia aryabhattai* | 802 | Fungi | *Cunninghamella elegans* |
| 692 | Bacteria | *Yokenella regensburgei* | 804 | Fungi | *Cunninghamella elegans* |
| 694 | Bacteria | *Yokenella regensburgei* | 807 | Fungi | *Mortierella globalpina* |
| 695 | Bacteria | *Priestia aryabhattai* | 812 | Fungi | *Penicillium raistrickii* |
| 697 | Bacteria | *Priestia megaterium* | 813 | Fungi | *Cunninghamella elegans* |
| 699 | Bacteria | *Yokenella regensburgei* | 814 | Fungi | *Aspergillus deflectus* |
| 702 | Bacteria | *Yokenella regensburgei* | 818 | Fungi | *Trichoderma neokoningii* |
| 703 | Bacteria | *Pseudomonas sp.* | 819 | Fungi | *Talaromyces atroroseus* |
| 706 | Bacteria | *Yokenella regensburgei* | 822 | Fungi | *Mortierella globalpina* |
| 707 | Bacteria | *Yokenella regensburgei* | 824 | Fungi | *Trichoderma yunnanense* |
| 708 | Bacteria | *Yokenella regensburgei* | 826 | Fungi | *Talaromyces atroroseus* |
| 709 | Bacteria | *Priestia aryabhattai* | 827 | Fungi | *Mortierella globalpina* |
| 713 | Bacteria | [*Yokenella regensburgei*](https://www.ncbi.nlm.nih.gov/Taxonomy/Browser/wwwtax.cgi?id=158877) | 828 | Fungi | *Cunninghamella elegans* |
| 715 | Bacteria | *Paenibacillus triticicola* | 829 | Fungi | *Talaromyces atroroseus* |
| 717 | Bacteria | *Priestia megaterium* | 830 | Fungi | *Talaromyces atroroseus* |
| 718 | Bacteria | *Rouxiella badensis* | 832 | Fungi | *Albifimbria viridis* |
| 719 | Bacteria | *Priestia aryabhattai* | 833 | Fungi | *Mortierella globalpina* |
| 720 | Bacteria | *Yokenella regensburgei* | 835 | Fungi | *Mortierella globalpina* |
| 721 | Bacteria | *Yokenella regensburgei* | 838 | Fungi | *Penicillium sp.* |
| 722 | Bacteria | *Calidifontibacillus erzurumensis* | 845 | Fungi | *Penicillium virgatum* |
| 723 | Bacteria | *Pseudomonas uvaldensis* | 848 | Fungi | *Mortierella globalpina* |
| 724 | Bacteria | *Calidifontibacillus erzurumensis* | 849 | Fungi | *Trichoderma yunnanense* |
| 725 | Bacteria | [*Yokenella regensburgei*](https://www.ncbi.nlm.nih.gov/Taxonomy/Browser/wwwtax.cgi?id=158877) | 851 | Fungi | *Trichoderma neokoningii* |
| 726 | Bacteria | *Priestia megaterium* | 852 | Fungi | *Plectosphaerella cucumerina* |
| 727 | Bacteria | *Peribacillus frigoritolerans* | 854 | Fungi | *Mortierella globalpina* |
| 728 | Bacteria | *Priestia megaterium* | 856 | Fungi | *Penicillium sp.* |
| 729 | Bacteria | *Calidifontibacillus erzurumensis* | 857 | Fungi | *Chaetomium subglobosum* |
| 731 | Bacteria | *Calidifontibacillus erzurumensis* | 858 | Fungi | *Mortierella globalpina* |
| 732 | Bacteria | *Yokenella regensburgei* | 976 | Fungi | *Mortierella globalpina* |
| 734 | Bacteria | *Yokenella regensburgei* | 982 | Fungi | *Mortierella globalpina* |
| 735 | Bacteria | *Yokenella regensburgei* | 983 | Fungi | *Mortierella globalpina* |
| 737 | Bacteria | *Yokenella regensburgei* | 984 | Fungi | *Mortierella globalpina* |
| 738 | Bacteria | *Calidifontibacillus erzurumensis* | 989 | Fungi | *Mortierella globalpina* |
| 1052 | Bacteria | *Calidifontibacillus erzurumensis* | 990 | Fungi | *Mortierella globalpina* |
| 1057 | Bacteria | *Brevibacillus laterosporus* | 992 | Fungi | *Penicillium glaucoroseum* |
| 1061 | Bacteria | *Calidifontibacillus erzurumensis* | 993 | Fungi | *Trichoderma yunnanense* |
| 1064 | Bacteria | *Calidifontibacillus erzurumensis* | 996 | Fungi | *Penicillium sp.* |
| 1070 | Bacteria | *Calidifontibacillus erzurumensis* | 997 | Fungi | *Mortierella globalpina* |
| 1071 | Bacteria | *Calidifontibacillus erzurumensis* | 999 | Fungi | *Mortierella globalpina* |
| 1073 | Bacteria | *Pseudomonas sp.* |  |  |  |
